# Tailored risk assessment of 90‐day acute heart failure readmission or all‐cause death to heart failure with preserved versus reduced ejection fraction

**DOI:** 10.1002/clc.23780

**Published:** 2022-01-25

**Authors:** Jaeyoung Park, Xiang Zhong, Farnaz Babaie Sarijaloo, Anita Wokhlu

**Affiliations:** ^1^ Department of Industrial and Systems Engineering University of Florida Gainesville Florida USA; ^2^ Division of Cardiovascular Medicine University of Florida Gainesville Florida USA

**Keywords:** congestive heart failure, HFpEF, machine learning, readmission

## Abstract

**Background:**

After incident heart failure (HF) admission, patients are vulnerable to readmission or death in the 90‐day post‐discharge. Although risk models for readmission or death incorporate ejection fraction (EF), patients with HF with preserved EF (HFpEF) and those with HF with reduced EF (HFrEF) represent distinct cohorts. To better assess risk, this study developed machine learning models and identified risk factors for the 90‐day acute HF readmission or death by HF subtype.

**Methods and Results:**

Approximately 1965 patients with HFpEF and 1124 with HFrEF underwent an index admission. Acute HF rehospitalization or death occurred in 23% of HFpEF and 28% of HFrEF groups. Of the 101 variables considered, multistep variable selection identified 24 and 25 significant factors associated with 90‐day events in HFpEF and HFrEF, respectively. In addition to risk factors common to both groups, factors unique to HFpEF patients included cognitive dysfunction, low‐pulse pressure, β‐blocker, and diuretic use, and right ventricular dysfunction. In contrast, factors unique to HFrEF patients included a history of arrhythmia, acute HF on presentation, and echocardiographic characteristics like left atrial dilatation or elevated mitral E/A ratio. Furthermore, the model tailored to HFpEF (area under the curve [AUC] = 0.770; 95% confidence interval [CI] 0.767–0.774) outperformed a model for the combined groups (AUC = 0.759; 95% CI 0.756–0.763).

**Conclusion:**

The UF 90‐day post‐discharge acute HF Re admission or Death Risk Assessment (UF90‐RADRA) models help identify HFpEF and HFrEF patients at higher risk who may require proactive outpatient management.

**Abbreviations**
BMI: body mass index
BP: blood pressure
BUN: blood urea nitrogen
EF: ejection fraction
EHR: electronic health record
HF: heart failure
HFpEF: heart failure with preserved ejection fraction
HFrEF: heart failure with reduced ejection fraction
LV: left ventricular
MAP: mean arterial pressure
NT‐Pro BNP: N‐terminal pro brain natriuretic peptide

## 1 INTRODUCTION

Readmission or death soon after an index heart failure (HF) admission is a frequent problem occurring in nearly one in four HF patients within 30 days after discharge.^1^ Furthermore, this period of increased vulnerability after an incident HF hospitalization may last up to 90 days post discharge.^2^, ^3^ According to a study from the National Readmission Database, 18% of HF encounters had a readmission within 30 days, while 31% of encounters had a readmission within 90 days.^4^ Because of the substantial economic burden of early HF readmission and the opportunity to proactively intervene in higher‐risk patients, there is strong impetus to identify patients at heightened risk of readmission or death soon after hospitalization. However, current post‐HF discharge risk prediction models are limited by their only moderately predictive capabilities.^5^, ^6^, ^7^ One potential reason for the suboptimal performance of such models may be the failure to separately consider distinct HF phenotypic subtypes—specifically those patients with heart failure with preserved ejection fraction (HFpEF) versus those with heart failure with reduced ejection fraction (HFrEF).

HFpEF is an HF subtype that identifies patients with relatively preserved systolic function—variably defined, but often with left‐ventricular ejection fraction (LVEF) ≥ 50%. Previous HF admission portends a greater increased long‐term risk of readmission or death in HFpEF patients compared with HFrEF patients.^8^ However, there are few studies focusing on early post‐discharge risk prognostication specific to the HFpEF or HFrEF population.^9^, ^10^, ^11^, ^12^, ^13^ Although HFpEF patients experience HF hospitalization and short‐term readmission at rates similar to HFrEF patients, some critical risk factors may be distinct from HFrEF patients.^14^, ^15^, ^16^ Demographically, HFpEF patients tend to be older and more often female; clinically, they share a higher burden of noncardiac comorbidities, demonstrate different echocardiographic findings, and require different pharmacological treatment.^17^ Previous studies have not fully exploited the gamut of data available within the electronic health record (EHR). For these reasons, there is interest in developing a unique risk stratification schema to identify hospitalized HFpEF patients at increased risk of post‐discharge acute HF readmission or all‐cause death.

In this study, we developed the UF 90‐day post‐discharge acute HF Readmission or Death Risk Assessment (UF90‐RADRA) models tailored to HF subtype. Building upon our previous effort to develop a generic HF risk prediction model, we improved the study design by incorporating more variables and developing models specific to HFpEF and HFrEF subtypes with the goal of identifying patients at elevated 90‐day risk of acute HF readmission or all‐cause mortality at the time of discharge.^18^ Identifying such high‐risk patients at the point of discharge is critical to anticipating the need for additional support for these patients.

## 2 METHODS

### 2.1 Patient population

This retrospective study was approved by the University of Florida (UF) Institutional Review Board and Privacy Office as an exempt study with a waiver of informed consent. Using EPIC EHR, we previously identified a cohort of 3189 local patients with established outpatient primary care or cardiology care at UF Shands who underwent an incident HF hospitalization (i.e., their primary or secondary diagnosis was HF identified by ICD‐9 or ICD‐10 codes) between January 2011 and January 2019 and survived to discharge. Inclusion and exclusion criteria, including qualifying ICD codes, were described previously.^18^ For this unique analysis, patients were classified according to HF subtypes, as either having HFpEF (LVEF ≥ 50%) or HFrEF (LVEF < 50%), based on ejection fraction.

### 2.2 Outcomes

For each subtype, the primary outcome was assessed. The primary outcome was a composite outcome of acute HF readmission or all‐cause mortality within 90 days after discharge from an index HF hospitalization. Acute HF readmission was identified by any of the following: (1) primary HF diagnosis, (2) secondary HF diagnosis denoting acuity (with previously described diagnostic codes) or (3) secondary HF diagnostic codes of any acuity along with documentation of intravenous diuretic administration.^18^ In patients who experienced multiple events within the 90‐day window such as HF readmission and death, only the first event was counted.

### 2.3 Data extraction

Using the EPIC EHR and the McKesson Change echocardiography reporting system, ten categories of variables were considered: (1) demographic and socioeconomic characteristics, (2) outpatient care characteristics, (3) social history including lifestyle and noncompliance behavior, (4) cardiovascular history, (5) other medical history, (6) hospitalization characteristics, (7) laboratory characteristics including cardiac biomarkers, (8) vitals, (9) medications, and (10) comprehensive transthoracic echocardiographic findings. For missing echocardiographic data, an experienced physician reader provided blinded interpretation based on accepted standards.^19^, ^20^

Both models incorporated 101 input variables that had at least 50% data in available subjects and sufficient heterogeneity to support a regression model.^21^ Among them, 91 variables had < 1.0% missing data. Six variables (LA size, E/e' ratio, peak E wave, e' velocity, E/a ratio, and troponin t) had 1%–25% missing data while four variables (peak A wave, albumin, bicarbonate, and NT proBNP) had 26%–49% missing data. Missing values were imputed for each group using Multivariate Imputation by Chained Equations.^22^ According to the guidance regarding multiple imputations,^23^ we expected to obtain unbiased results even with the variables that have large proportions of missing data, given data are missing at random. To validate this assumption, we performed sensitivity analyses by using different imputed values and demonstrated the robustness of our models, that is, varying the imputed values does not affect the major results of the prediction models.

### 2.4 Variable selection

To develop models specific to the HFpEF and HFrEF subtypes, the variable selection was performed in parallel for each HF subtype. Variable selection is an important step to ensure a robust and efficient predictive model so we employed a multistep strategy with machine learning (ML) techniques.^24^ First, univariate analysis was performed. A less strict threshold for *p*‐value was employed to consider potential joint associations between multiple variables and the outcome. Using the *t*‐test for continuous variables and the chi‐squared test for categorical variables, candidate univariates with *p* < .10 were included in a penalized logistic regression (LR) model with the least absolute shrinkage and selection operator (LASSO) penalty, for further selection. Using the LASSO LR model and four‐fold cross‐validation, variables selected in at least three out of four folds were chosen for developing a standard LR model without penalty for ease of interpretation, i.e., estimating the odds ratio of risk factors. The stepwise selection process based on Akaike Information Criterion was applied to increase efficiency for estimating significant risk factors.

### 2.5 Model performance

After the variable selection, a final standard LR model was developed for each subtype, and we obtained the probability (a risk score) from each model. For classification, Youden's J‐statistic^25^ was used to determine the decision rule (i.e., a cutoff of the risk score) to predict which patient will be readmitted due to acute HF or die within 90 days. For model performance, we measured the area under the receiver operating characteristic curve (AUC), accuracy, sensitivity, and specificity for each model. To avoid overfitting the prediction models, we used repeated four‐fold cross‐validation (i.e., repeatedly building a model using 75% of the data randomly sampled and evaluating the model performance using the remaining 25%).

### 2.6 Risk factor analysis

Odds ratios were used to determine whether a particular variable is a risk factor for the outcome of 90‐day acute HF readmission or death, and to compare the magnitude of various risk factors for the outcome. In the standard LR model, coefficients of variables with *p* < .05 were considered as significant. In addition, to ensure the models are robust to missing values, the results with different imputed missing values were compared to identify any impact on model performance.

### 2.7 Software

Analyses were performed using R version 3.6.0^26^ and R packages including glmnet^27^ and pROC.^28^

## 3 RESULTS

### 3.1 Clinical outcomes

**Figure 1:**
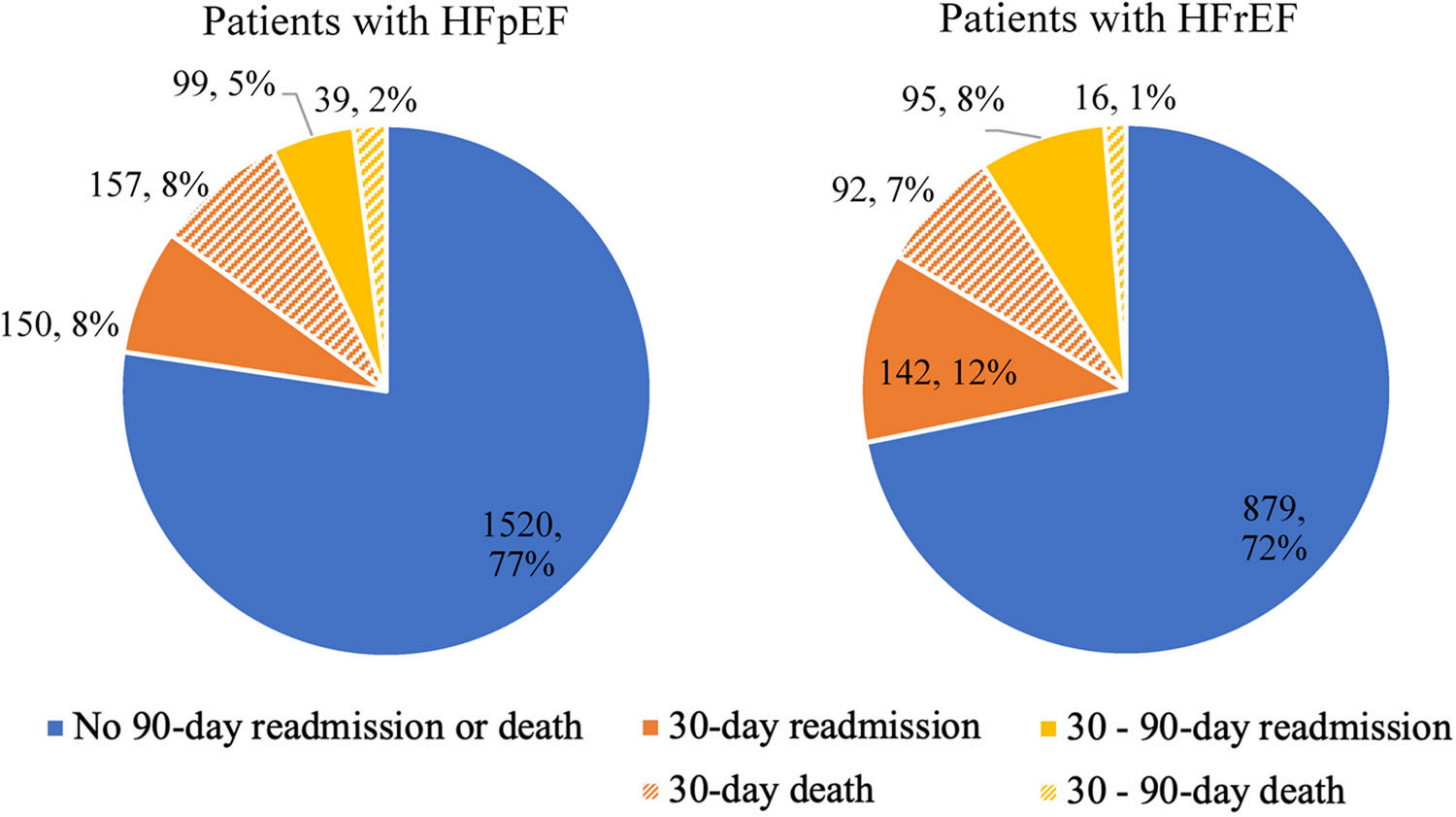
Endpoints based on the HF subtypes. HFpEF, heart failure with preserved ejection fraction; HFrEF, heart failure with reserved ejection fraction

Of the 3189 patients discharged from index HF admission, we identified 1965 (62%) with HFpEF and 1224 (38%) with HFrEF. Figure 1 demonstrates the timing of post‐discharge events in each group. Among the HFpEF group, 445 (23%) experienced either HF readmission or death within 90 days whereas, among those with HFrEF, 345 (28%) experienced the 90‐day HF composite outcome. HF readmission or death at 90‐days occurred more frequently in the HFrEF subgroup (*p* < .001).

### 3.2 Patient and care characteristics

Comprehensive characteristics for all 101 variables across 10 categories are compared for these two groups in Table S1. To summarize, in the HFpEF group, the average LVEF was 59.9 ± 5.4%, whereas in the HFrEF group, the LVEF was 29.9 ± 10.1% (*p* < .001). HFpEF patients, on average, were older, more likely to be female, and demonstrated more non‐cardiovascular comorbidities, in general, especially sleep apnea or lung disease. In contrast, HFrEF patients had a higher incidence of coronary artery disease, presented more acutely and with a higher average NT pro‐BNP, demonstrated more right ventricular dysfunction, and were more often prescribed various cardiovascular medications.

### 3.3 Risk factors identification based on HF subtypes

**Figure 2:**
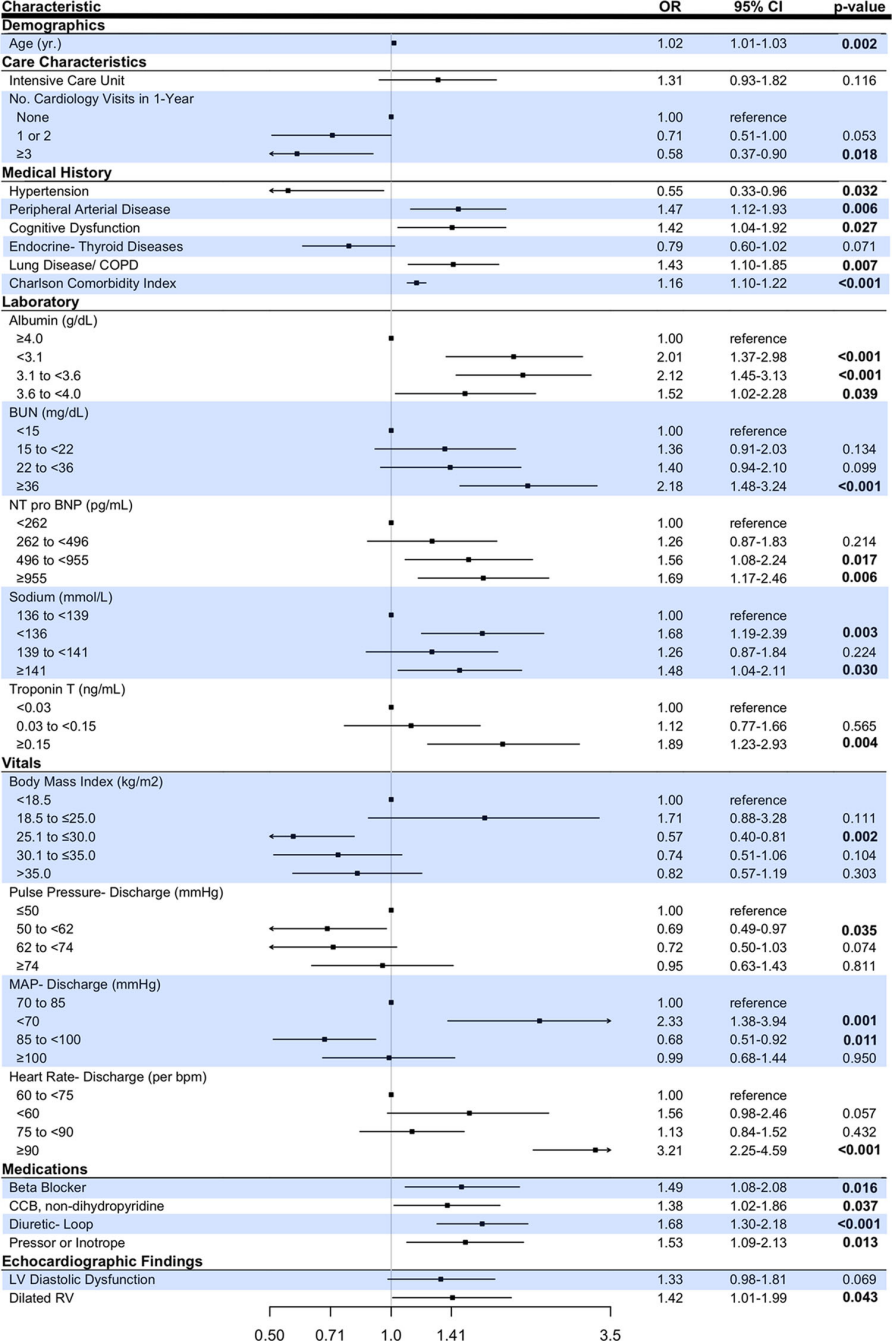
Forest plot of the risk factors for the HFpEF subtype associated with 90‐day acute heart failure or all‐cause death. An odds ratio (OR) and its 95% confidence interval (CI) for each characteristic are displayed in the middle. A *p* value less than .05 indicates a significant risk factor and is bold. BP, blood pressure; bpm, beats per minute; BUN, blood urea nitrogen; CI, confidence interval; COPD, chronic obstructive pulmonary disease; HFpEF, heart failure with preserved ejection fraction; LV, left ventricular; MAP, mean arterial pressure; MV, mitral valve; NT pro‐BNP, N terminal pro‐brain natriuretic peptide; OR, odds ratio; RV, right ventricular

**Figure 3:**
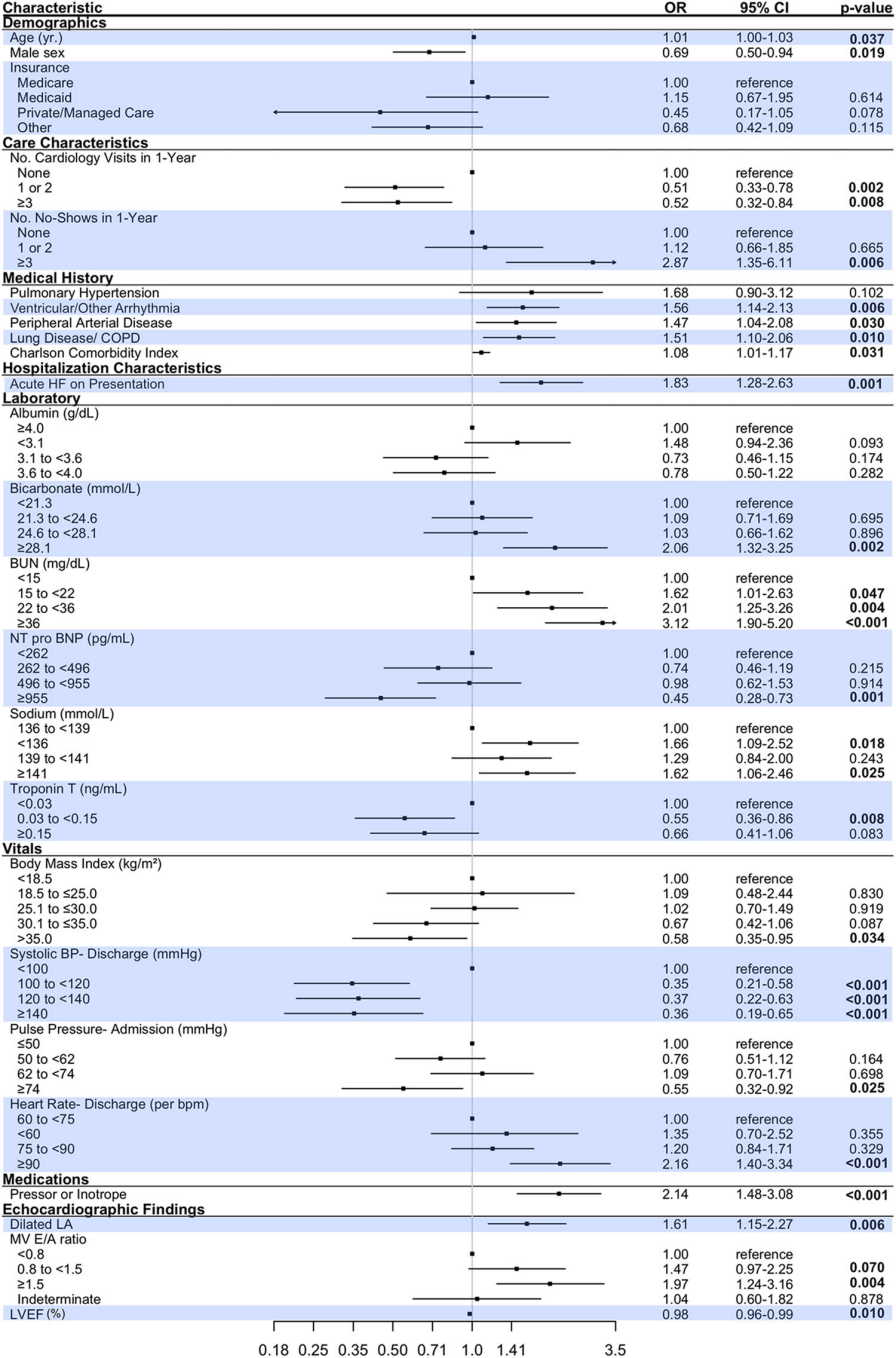
Forest plot of the risk factors for the HFrEF subtype associated with 90‐day acute heart failure or all‐cause death. BP, blood pressure; bpm, beats per minute; BUN, blood urea nitrogen; CI, confidence interval; COPD, chronic obstructive pulmonary disease; HFrEF, heart failure with reserved ejection fraction; LV, left ventricular; MAP, mean arterial pressure; MV, mitral valve; NT pro‐BNP, N‐terminal pro‐brain natriuretic peptide; OR, odds ratio; RV, right ventricular

Univariate analysis was performed for all 101 patient and care characteristics (variables) as part of the variable selection process, as shown in Table S2 (HFpEF) and Table S3 (HFrEF). In the HFpEF group, 59 variables with a *p* < .10 were included in the LASSO LR model, whereas in the HFrEF group, 58 variables meeting this criterion were included. In the next step, the LASSO LR models picked 35 and 39 variables for the HFpEF and the HFrEF subtype, respectively. In the final standard LR models specific to each subtype, 24 characteristics were identified as significant in the HFpEF patients, as shown in Figure 2, and 25 characteristics were identified as significant in the HFrEF patients, as shown in Figure 3.

**Figure 4:**
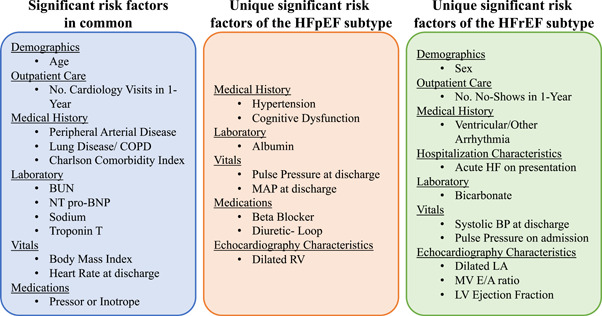
Significant risk factors based on the subtypes. BP, blood pressure; BUN, blood urea nitrogen; COPD, chronic obstructive pulmonary disease; HFpEF, heart failure with preserved ejection fraction; HFrEF, heart failure with reserved ejection fraction; LV, left ventricular; MAP, mean arterial pressure; MV, Mitral Valve; NT pro‐BNP, N‐terminal pro‐brain natriuretic peptide; RV, right ventricular

Figure 4 highlights the significant variables common to and unique to HF subtypes. Based on the magnitude of the odds ratio, the strongest common risk factors to both models appeared to be laboratory characteristics, including elevated BUN, NT pro‐BNP, Troponin T, and low sodium, as well as requiring taking pressors or inotropic therapy on the index admission. Risk factors with the highest odds ratios specific to the HFpEF subtype included low albumin, low MAP at discharge, and requiring loop diuretics. For the HFrEF subtype, the risk factors that were more predictive of adverse HF outcomes included a history of arrhythmia, acute HF on presentation, and echocardiographic characteristics like left atrial dilatations or elevated mitral E/A ratio.

### 3.4 Models of readmission risk by HF subtype

Using these models, we identified optimal cutoff scores as indicative of elevated 90‐d readmission and mortality risk. The performances of the HFpEF, HFrEF, and the generic or combined HF models are shown in Table 1. The average AUC of the HFpEF model was 0.770 (95% CI 0.767–0.774) with incrementally better performance characteristics including improved sensitivity when compared with the generic model, despite representing a smaller subgroup of the cohort. Although the HFrEF model had a slightly lower AUC of 0.755 (0.750, 0.761), its accuracy and specificity were >70% and its sensitivities were 64.1% (63.3%, 64.8%) simultaneously. We collectively refer to these HF‐type‐specific models as the UF90‐RADRA decision support tools for HFpEF or HFrEF.

**Table 1:** UF‐90 RADRA models’ performance based on readmission risk scores in HFpEF versus HFrEF

Performance metrics	HFpEF (average; 95% CI)	HFrEF (average; 95% CI)	Generic HF cohort (average; 95% CI)
AUC	0.770 (0.767–0.774)	0.755 (0.750–0.761)	0.759 (0.756–0.763)
Optimal cutoff	0.243	0.297	0.271
Accuracy	71.9% (71.4%–72.2%)	70.3% (69.8%–70.8%)	71.6% (71.3%–71.8%)
Sensitivity	67.0% (66.3%–67.8%)	64.9% (63.8%–66.0%)	64.1% (63.3%–64.8%)
Specificity	73.3% (72.8%–73.9%)	72.5% (71.9%–73.2%)	74.0% (73.7%–74.4%)

*Note*: The optimal cutoff was obtained using all the patients for each subtype, and the models were evaluated with four‐fold cross‐validation.

Abbreviations: AUC, area under the receiver operating characteristic; CI, confidence interval; HFpEF, heart failure with preserved ejection fraction; HFrEF, heart failure with reserved ejection fraction.

## 4 DISCUSSION

Combining HFpEF and HFrEF patients to develop generic post‐discharge HF risk prediction tools overlooks the fundamental differences between these already heterogeneous populations. The UF90‐RADRA models for HFpEF and HFrEF are unique postdischarge HF risk assessment tools in a few ways. First, they generate risk information from the 90‐day post‐discharge vantage point, with a focus on acute HF readmission and death, which is a clinically relevant outcome that would motivate cardiology providers to more carefully monitor and manage such patients. In addition, these tools are uniquely tailored to assess risk specific to HF subtype, with algorithm that can be potentially incorporated within an EHR. Finally, they demonstrate favorable performance characteristics despite the known difficulties in HF modeling (AUC > 0.75). In this study, we identified thirty shared or unique risk factors for 90‐day acute HF readmission or all‐cause death. The complex array of risk factors specific to HF subtype underscores the potential advantages of clinical support tools like the UF90‐RADRA over provider intuition to screen for high‐risk HF patients.

### 4.1 Risk prediction specific to HF subtype

We are not aware of other 90‐day post‐discharge risk prediction tools specific to HF subtypes. Existing HF discharge tools focus on 30‐day outcomes.^7^, ^11^, ^14^ Although there is obvious value in 30‐day post‐discharge risk prediction from a reimbursement perspective, the window of vulnerability for heightened risk of readmission or death likely lasts up to 90 days before patients return to their normative states.^29^ In our cohort, for example, ~10% of patients experienced acute HF readmission or death at 31–90 days post‐discharge. Events in this time frame may still reflect gaps in postdischarge care that require attention.

Beyond the distinct timing aspect of our risk prediction tools, the main advantage of the UF90‐RADRA models is that they are specific to HFpEF and HFrEF. At least ten unique characteristics in each HF subtype, as well as several overlapping risk characteristics were identified, reinforcing the value of tailored post‐discharge risk prediction tools.

### 4.2 Model performance in context

Another important feature of the UF90‐RADRA models for HFpEF and HFrEF is their robust performance. The HFpEF and HFrEF model demonstrated AUC's of approximately 0.77 and approximately 0.76, respectively. In addition, both models demonstrated an accuracy >70%, a sensitivity >60%, and a specificity>70% simultaneously. This is a favorable performance based on the reported performance of existing HF risk prediction tools. However, direct comparison of these models is not feasible given the narrow array of available risk prediction tools. The most widely used HF risk prediction tool for hospitalized patients is the 30‐day Yale readmission risk score.^30^ It has the advantage of having been validated or re‐assessed in multiple cohorts. However, it has a modest predictive ability with an approximate AUC of approximately 0.6, does not specify HF subtypes, and is intended for use in patients aged 65 years and older.^31^ Unlike our models, the Yale score focuses on all‐cause readmission risk and analyzes data available at the time of admission. In comparison, the UF90‐RADRA decision support tools are ideally used at the point of discharge—characterizing patients in a binary fashion as normal versus elevated risk for the combined endpoint of 90‐day acute HF readmission or death. By focusing on higher acuity HF endpoints, the algorithm's classification of elevated risk justifies aggressive post‐discharge cardiovascular HF care.

In particular, previous studies of risk prediction in HFpEF have relied heavily on existing generic risk prediction tools—some not even specific to HF patients—incorporating a few additional characteristics with unfavorable results.^10^, ^16^ For example, a single‐center study looking at the HOSPITAL Score, LACE index, and LACE + index in HFpEF patients concluded that they are not effective predictors of 30‐day readmission for patients with HFpEF.^6^ More elaborate risk prediction in HFpEF has looked at longer‐term outcomes, that is, >1‐year and includes some variables not routinely obtained in HF patients such as fibrosis indicators on cardiac MRI or functional testing.^12^, ^32^ By focusing on the extensive and readily available data elements extractable from the EHR, the UF90‐RADRA risk assessment tools have the potential for development for real‐time integration within the EHR.

### 4.3 Risk factors in HFpEF versus HFrEF

In this study, potential mediators of risk for each HF subtype were identified from over 100 candidate variables from the EHR using ML‐based variable selection—yielding new insights into the profile of HF patients at elevated risk. The risk factors are common to both HF subtypes associated with 90‐day acute HF readmission or all‐cause death highlight the negative impact of serious comorbidity, such as advanced lung disease or multiple comorbidities as described by a high Charlson comorbidity index. In addition, laboratories generally associated with greater HF severity including elevated cardiac biomarkers including troponin and NT pro‐BNP or abnormal sodium or renal function were associated with the 90‐day composite event. In HFpEF patients, low mean arterial pressure, albumin, presumably a marker of nutritional status or liver disease, and the recent use of diuretics were associated with increased 90‐day post‐discharge events. In contrast, for HFrEF patients, a variety of echocardiographic variables including LVEF and some diastolic parameters were more relevant to outcome.

### 4.4 Limitations

There are some limitations to our study. First, this was a single‐center, retrospective study that required data extraction from the EHR. Furthermore, although multiple imputations is a standard method to address missing data, it may introduce bias. To address this, we performed sensitivity analyses and saw no significant impact, as summarized in Table S4. In addition, the models were developed from groups between 1000 and 2000 patients. In order for the UF90‐RADRA models to be a valuable clinical tool, efforts should be made to prospectively validate and optimize their performance using larger datasets across sites.

## 5 CONCLUSION

The UF90‐RADRA models for HFpEF and HFrEF have potential as unique tools in the armamentarium for early post‐discharge HF risk assessment. The complexity of the models and the number of risk factors within each underscore that even a seasoned clinician may have difficulty identifying patients at elevated postdischarge risk on his/her own. The relatively strong performance characteristics of these models using modest size cohorts highlight the value of EHR extraction techniques and advanced statistical methodologies which include machine learning. Future studies optimizing and validating UF90‐RADRA risk assessment tool, particularly in larger HFpEF cohorts, are needed—with the ultimate goal of EHR integration.

## CONFLICT OF INTERESTS

The authors declare that there are no conflict of interests.

## Supporting information

Supplementary information.

Supplementary information.

Supplementary information.

Supplementary information.

## Data Availability

The data used in this study are not open to public and the approval from the University of Florida Institutional Review Board and Privacy Office is required.
