# Supplementary material for: Tailored risk assessment of 90‐day acute heart failure readmission or all‐cause death to heart failure with preserved versus reduced ejection fraction
Source: Clin Cardiol. 2022 Jan 25;45(4):370–8. doi: 10.1002/clc.23780 (PMC9019897; doi:10.1002/clc.23780)
Supplement: Supplementary file 1 — Supplementary information. [file CLC-45-370-s001.docx]

**Supplemental Table 1. Characteristics based on the HF subtypes.**

| **Characteristic** | **HFpEF (N=1965)** | **HFrEF**  **(N=1224)** | **P-value^[[1]](#endnote-1)^** |
| --- | --- | --- | --- |
| **Demographic & Socioeconomic** | | | |
| Age (yr.) | 69.3 ± 14.8 | 65.9 ± 15.0 | **<0.001** |
| Male sex | 756 (38.5) | 713 (58.3) | **<0.001** |
| Race |  |  | 0.124 |
| *Black* | 572 (29.1) | 379 (31.0) |  |
| *White* | 1326 (67.5) | 790 (64.5) |  |
| *Other* | 67 (3.4) | 55 (4.5) |  |
| Married | 747 (38.0) | 512 (41.8) | **0.035** |
| Rural Resident | 1746 (88.9) | 1070 (87.4) | 0.242 |
| Insurance |  |  | **<0.001** |
| *Medicare* | 1454 (74.0) | 765 (62.5) |  |
| *Medicaid* | 180 (9.2) | 151 (12.3) |  |
| *Private/Managed Care* | 63 (3.2) | 54 (4.4) |  |
| *Other* | 268 (13.6) | 254 (20.8) |  |
| **Outpatient Care** | | | |
| Electronic Health Portal Use | 488 (24.8) | 276 (22.5) | 0.153 |
| No. Cardiology Visits in 1-Year | 0 (0, 1) | 0 (0, 1) | **0.002** |
| No. No-Shows in 1-Year | 0 (0, 0) | 0 (0, 0) | **0.003** |
| **Social History** | | | |
| Tobacco Abuse/Smoking | 1031 (52.5) | 720 (58.8) | **0.001** |
| Alcohol Dependence | 217 (11.0) | 128 (10.5) | 0.646 |
| Illicit Drug Use | 186 (9.5) | 86 (7.0) | **0.020** |
| Noncompliance^[[2]](#endnote-2)^ | 632 (32.2) | 342 (27.9) | **0.013** |
| **Medical History (Cardiovascular-related)** | | | |
| Hypertension | 1850 (94.1) | 1119 (91.4) | **0.004** |
| Dyslipidemia | 1531 (77.9) | 878 (71.7) | **<0.001** |
| Pulmonary Hypertension | 187 (9.5) | 66 (5.4) | **<0.001** |
| Cardiomyopathy Diagnosis | 482 (24.5) | 760 (62.1) | **<0.001** |
| Congestive Heart Failure | 1119 (56.9) | 674 (55.1) | 0.315 |
| Coronary Artery Disease | 1142 (58.1) | 795 (65.0) | **<0.001** |
| Myocardial Infarction | 470 (23.9) | 434 (35.5) | **<0.001** |
| CABG or PCI | 907 (46.2) | 614 (50.2) | **0.030** |
| CVA/TIA/Intracranial | 1152 (58.6) | 615 (50.2) | **<0.001** |
| Pacemaker or Defibrillator | 117 (6.0) | 179 (14.6) | **<0.001** |
| Valvular Heart Disease | 718 (36.5) | 370 (30.2) | **<0.001** |
| Atrial Arrhythmia | 914 (46.5) | 607 (49.6) | 0.098 |
| Ventricular/Other Arrhythmia | 1187 (60.4) | 698 (57.0) | 0.064 |
| Peripheral Arterial Disease | 588 (29.9) | 313 (25.6) | **0.009** |
| **Medical History (Other)** |  |  |  |
| Moderate/Severe Renal Disease | 612 (31.1) | 345 (28.2) | 0.083 |
| Malignancy/Cancer | 1267 (64.5) | 683 (55.8) | **<0.001** |
| Depression/Psychiatric | 1190 (60.6) | 616 (50.3) | **<0.001** |
| Cognitive Dysfunction | 389 (19.8) | 181 (14.8) | **<0.001** |
| Diabetes | 1069 (54.4) | 612 (50.0) | **0.017** |
| Endocrine- Thyroid Diseases | 727 (37.0) | 344 (28.1) | **<0.001** |
| Hypogonadism | 220 (11.2) | 87 (7.1) | **<0.001** |
| Venous Thromboembolism | 309 (15.7) | 144 (11.8) | **0.002** |
| Liver Disease | 557 (28.3) | 291 (23.8) | **0.005** |
| Sleep Apnea/ Disorder | 843 (42.9) | 390 (31.9) | **<0.001** |
| Lung Disease/COPD | 1154 (58.7) | 612 (50.0) | **<0.001** |
| Chronic Oxygen Use | 311 (15.8) | 109 (8.9) | **<0.001** |
| Charlson Comorbidity Index | 3.9 ± 2.2 | 3.6 ± 2.1 | **<0.001** |
| **Hospitalization Characteristics** |  |  |  |
| Acute HF on Presentation^^[[3]](#endnote-3)^^ | 1055 (53.7) | 788 (64.4) | **<0.001** |
| Length of Stay (days) | 7.2 ± 11.1 | 7.5 ± 15.5 | 0.630 |
| Observation Status | 1807 (92.0) | 1139 (93.1) | 0.286 |
| Intensive Care Unit | 424 (21.6) | 307 (25.1) | **0.025** |
| Discharge Med Reconciliation | 226 (11.5) | 115 (9.4) | 0.070 |
| **Laboratory** |  |  |  |
| Albumin (g/dL) | 3.8 (3.5, 4.2) | 3.9 (3.5, 4.2) | **0.010** |
| Bicarbonate (mmol/L**)** | 25.3 (22.7, 28.6) | 25.0 (22.0, 27.7) | **<0.001** |
| BUN (mg/dL) | 20.0 (14.5, 29.9) | 19.7 (14.8, 29.2) | 0.483 |
| Creatinine (mg/dL) | 1.0 (0.8, 1.5) | 1.1 (0.8, 1.4) | **0.012** |
| Hemoglobin (g/dL) | 11.1 (9.5, 12.6) | 11.9 (10.1, 13.5) | **<0.001** |
| NT pro-BNP (pg/mL) | 413 (225, 828) | 542 (285, 990) | **<0.001** |
| Sodium (mmol/L) | 139 (137, 141) | 139 (137, 140) | 0.084 |
| Troponin T (ng/mL) | 0.0 (0.0, 0.0) | 0.0 (0.0, 0.1) | **<0.001** |
| **Vitals** |  |  |  |
| Body Mass Index (kg/m^2^) | 29.4 (24.5, 36.2) | 27.6 (23.7, 33.3) | **<0.001** |
| Weight (kg) |  |  |  |
| Weight Gain at Presentation | 0.0 (-3.0, 2.1) | 0.0 (-3.2, 1.2) | **0.004** |
| Weight Loss Over Hospitalization | 0.0 (-0.9, 2.0) | 0.0 (-0.6, 3.0) | **0.003** |
| Weight Loss from Maximum | 1.7 (0.0, 5.2) | 2.3 (0.0, 5.7) | **0.005** |
| Blood Pressure (mmHg) |  |  |  |
| Systolic BP- Admission | 135 (119, 152) | 129 (115, 147) | **<0.001** |
| Systolic BP- Discharge | 128 (115, 142) | 121 (108, 136) | **<0.001** |
| Diastolic BP- Admission | 70.6 (61.7, 80.0) | 74.8 (66.0, 85.0) | **<0.001** |
| Diastolic BP- Discharge | 69.0 (61.3, 77.2) | 70.7 (62.0, 78.7) | **0.024** |
| Pulse Pressure- Admission | 62.5 (50.5, 77.0) | 53.1 (42.8, 66.0) | **<0.001** |
| Pulse Pressure- Discharge | 58.3 (47.5, 70.0) | 49.4 (39.8, 61.0) | **<0.001** |
| MAP- Admission (mmHg) | 93.2 (83.4, 104.4) | 92.0 (82.2, 102.6) | 0.096 |
| MAP- Discharge (mmHg) | 86.9 (78.9, 96.9) | 88.5 (80.7, 98.0) | **0.009** |
| Heart Rate- Admission (mmHg) | 88.6 (75.0, 101.4) | 80.5 (70.0, 93.5) | **<0.001** |
| Heart Rate- Discharge (mmHg) | 77.0 (68.6, 86.5) | 75.5 (67.3, 85.0) | **0.044** |
| **Medications** | | | |
| Aspirin | 1353 (68.9) | 990 (80.9) | **<0.001** |
| ACE-I/ARB/ARNI | 1122 (57.1) | 937 (76.6) | **<0.001** |
| Aldosterone Antagonists | 115 (5.9) | 121 (9.9) | **<0.001** |
| Beta Blocker | 1516 (77.2) | 1149 (93.9) | **<0.001** |
| Antiarrhythmics | 243 (12.4) | 201 (16.4) | **0.002** |
| Anticoagulation | 478 (24.3) | 338 (27.6) | **0.043** |
| Ca-Channel Blocker (CCB), any | 738 (37.6) | 280 (22.9) | **<0.001** |
| CCB, non-dihydropyridine | 347 (17.7) | 206 (16.8) | 0.580 |
| Digoxin | 87 (4.4) | 81 (6.6) | **0.009** |
| Diuretic- Metolazone | 112 (5.7) | 79 (6.5) | 0.426 |
| Diuretic- Loop | 1026 (52.2) | 693 (56.6) | **0.017** |
| Diuretic- Thiazide | 325 (16.5) | 145 (11.8) | **<0.001** |
| Pressor or Inotrope | 429 (21.8) | 279 (22.8) | 0.554 |
| Nitrate | 239 (12.2) | 161 (13.2) | 0.443 |
| Hydralazine | 636 (32.4) | 288 (23.5) | **<0.001** |
| Statin | 1185 (60.3) | 852 (69.6) | **<0.001** |
| Insulin | 935 (47.6) | 592 (48.4) | 0.693 |
| Metformin | 144 (7.3) | 100 (8.2) | 0.423 |
| Any Estrogen | 34 (1.7) | 15 (1.2) | 0.328 |
| NSAID | 309 (15.7) | 158 (12.9) | **0.033** |
| **Echocardiographic Findings** |  |  |  |
| Dilated LV | 77 (3.9) | 260 (21.2) | **<0.001** |
| LV Diastolic Dysfunction | 1430 (72.8) | 1150 (94.0) | **<0.001** |
| LV Ejection Fraction | 59.9 ± 5.4 | 29.9 ± 10.1 | **<0.001** |
| Dilated LA | 983 (50.0) | 725 (59.2) | **<0.001** |
| Dilated RV | 252 (12.8) | 258 (21.1) | **<0.001** |
| RV Dysfunction | 177 (9.0) | 482 (39.4) | **<0.001** |
| Pulmonary Hypertension | 666 (33.9) | 399 (32.6) | 0.474 |
| Dilated Inferior Vena Cava | 131 (6.7) | 136 (11.1) | **<0.001** |
| Pericardial Effusion | 101 (5.1) | 64 (5.2) | 0.978 |
| MV E/A ratio | 1.0 (0.8, 1.3) | 1.1 (0.8, 1.6) | **<0.001** |
| *Indeterminate* | 321 (16.3) | 157 (12.8) |  |
| MV E/e' ratio | 14.0 (10.7, 18.8) | 15.8 (11.7, 21.8) | **<0.001** |
| MV Peak E Wave, cm/s | 97.4 ± 31.8 | 91.0 ± 30.3 | **<0.001** |
| MV Peak A Wave, cm/s | 88.5 ± 29.1 | 87.2 ± 307.7 | 0.992 |
| MV e' Velocity, cm/s | 6.9 ± 3.9 | 5.8 ± 2.6 | **<0.001** |

1. Comparisons between groups was performed using either a t-test or chi-square test. Variables with a p<0.05 (**bold**) were considered potentially significant and were selected further inclusion in machine-learning-based variable selection. [↑](#endnote-ref-1)
2. ICD-9/10 codes were used to identify noncompliance cases. [↑](#endnote-ref-2)
3. Acute HF on presentation is defined as either an ICD-9/10 code which denotes a secondary diagnosis of acute heart failure or an admission with a primary HF diagnosis or administration of intravenous diuretics during hospitalization with a secondary HF diagnostic codes of any acuity.

   ACE-I= angiotensin converting enzyme inhibitor; ARBs= angiotensin-receptor blockers; ARNI= angiotensin receptor-neprilysin inhibitor; BP= blood pressure; bpm= beats per minute; BUN= blood urea nitrogen; CABG= coronary artery bypass grafting; CCB= calcium channel blocker; COPD= chronic obstructive pulmonary disease; HF= heart failure; LV= left ventricular; MAP= mean arterial pressure; NSAID= non-steroidal anti-inflammatory drug; NT pro-BNP= N-terminal pro-brain natriuretic peptide; PCI= percutaneous coronary intervention; RV= right ventricle; TIA= transient ischemic attack. [↑](#endnote-ref-3)
