# Supplementary material for: Tailored risk assessment of 90‐day acute heart failure readmission or all‐cause death to heart failure with preserved versus reduced ejection fraction
Source: Clin Cardiol. 2022 Jan 25;45(4):370–8. doi: 10.1002/clc.23780 (PMC9019897; doi:10.1002/clc.23780)
Supplement: Supplementary file 2 — Supplementary information. [file CLC-45-370-s002.docx]

**Supplemental Table 2. Characteristics of HF with preserved EF based on 90-day acute heart failure readmission or all-cause death**

| **Characteristic** | **90-day HF event**  **(N=445)** | **No 90-day HF event**  **(N=1520)** | **P-value^[[1]](#endnote-1)^** |
| --- | --- | --- | --- |
| **Demographic & Socioeconomic** | | | |
| Age (yr.) | 72.1 ± 14.2 | 68.4 ± 14.9 | **<0.001** |
| Male sex | 177 (39.8) | 579 (38.1) | 0.558 |
| Race |  |  | 0.139 |
| *Black* | 113 (25.4) | 459 (30.2) |  |
| *White* | 315 (70.8) | 1011 (66.5) |  |
| *Other* | 17 (3.8) | 50 (3.3) |  |
| Married | 162 (36.4) | 585 (38.5) | 0.459 |
| Rural Resident | 40 (9.0) | 179 (11.8) | 0.119 |
| Insurance |  |  | **0.056** |
| *Medicare* | 350 (78.7) | 1104 (72.6) |  |
| *Medicaid* | 38 (8.5) | 142 (9.3) |  |
| *Private/Managed Care* | 10 (2.2) | 53 (3.5) |  |
| *Other* | 47 (10.6) | 221 (14.5) |  |
| **Outpatient Care** | | | |
| Electronic Health Portal Use | 108 (24.3) | 380 (25.0) | 0.802 |
| No. Cardiology Visits in 1-Year | 0 (0, 0) | 0 (0, 1) | **0.012** |
| No. No-Shows in 1-Year | 0 (0, 0) | 0 (0, 0) | 0.279 |
| **Social History** | | | |
| Tobacco Abuse/Smoking | 240 (53.9) | 791 (52.0) | 0.516 |
| Alcohol Dependence | 52 (11.7) | 165 (10.9) | 0.685 |
| Illicit Drug Use | 36 (8.1) | 150 (9.9) | 0.301 |
| Noncompliance^[[2]](#endnote-2)^ | 149 (33.5) | 483 (31.8) | 0.535 |
| **Medical History (Cardiovascular-related)** | | | |
| Hypertension | 411 (92.4) | 1439 (94.7) | **0.087** |
| Dyslipidemia | 339 (76.2) | 1192 (78.4) | 0.349 |
| Pulmonary Hypertension | 47 (10.6) | 140 (9.2) | 0.446 |
| Cardiomyopathy Diagnosis | 108 (24.3) | 374 (24.6) | 0.935 |
| Congestive Heart Failure | 252 (56.6) | 867 (57.0) | 0.921 |
| Coronary Artery Disease | 248 (55.7) | 894 (58.8) | 0.269 |
| Myocardial Infarction | 125 (28.1) | 345 (22.7) | **0.022** |
| CABG or PCI | 208 (46.7) | 699 (46.0) | 0.821 |
| Stroke or TIA | 271 (60.9) | 881 (58.0) | 0.293 |
| Pacemaker or Defibrillator | 30 (6.7) | 87 (5.7) | 0.494 |
| Valvular Heart Disease | 179 (40.2) | 539 (35.5) | **0.075** |
| Atrial Arrhythmia | 251 (56.4) | 663 (43.6) | **<0.001** |
| Ventricular/Other Arrhythmia | 292 (65.6) | 895 (58.9) | **0.012** |
| Peripheral Arterial Disease | 165 (37.1) | 423 (27.8) | **<0.001** |
| **Medical History (Other)** |  |  |  |
| Moderate/ Severe Renal Disease | 160 (36.0) | 452 (29.7) | **0.015** |
| Malignancy/ Cancer | 302 (67.9) | 965 (63.5) | 0.101 |
| Depression/ Psychiatric | 271 (60.9) | 919 (60.5) | 0.911 |
| Cognitive Dysfunction | 116 (26.1) | 273 (18.0) | **<0.001** |
| Diabetes | 236 (53.0) | 833 (54.8) | 0.545 |
| Endocrine- Thyroid Diseases | 149 (33.5) | 578 (38.0) | **0.091** |
| Hypogonadism | 48 (10.8) | 172 (11.3) | 0.821 |
| Venous Thromboembolism | 81 (18.2) | 228 (15.0) | 0.119 |
| Liver Disease | 139 (31.2) | 418 (27.5) | 0.139 |
| Sleep Apnea | 172 (38.7) | 671 (44.1) | **0.045** |
| Lung Disease/ COPD | 284 (63.8) | 870 (57.2) | **0.015** |
| Chronic Oxygen Use | 86 (19.3) | 225 (14.8) | **0.026** |
| Charlson Comorbidity Index | 4.6 ± 2.6 | 3.7 ± 2.0 | **<0.001** |
| **Hospitalization Characteristics** |  |  |  |
| Acute HF on Presentation^[[3]](#endnote-3)^ | 292 (65.6) | 763 (50.2) | **<0.001** |
| Length of Stay (days) | 9.7 ± 18.9 | 6.5 ± 7.3 | **<0.001** |
| Observation Status | 11 (2.5) | 147 (9.7) | **<0.001** |
| Intensive Care Unit | 141 (31.7) | 283 (18.6) | **<0.001** |
| Discharge Med Reconciliation | 54 (12.1) | 172 (11.3) | 0.695 |
| **Laboratory** |  |  |  |
| Albumin (g/dL) | 3.6 (3.1, 3.9) | 3.7 (3.3, 4.1) | **<0.001** |
| Bicarbonate (mmol/L**)** | 25.4 (22.0, 28.6) | 25.1 (22.7, 28.4) | **0.060** |
| BUN (mg/dL) | 26.2 (16.8, 37.9) | 18.8 (14.0, 27.2) | **<0.001** |
| Creatinine (mg/dL) | 1.1 (0.8, 1.9) | 1.0 (0.8, 1.4) | **<0.001** |
| Hemoglobin (g/dL) | 10.4 (9.0, 11.7) | 11.4 (9.8, 12.8) | **<0.001** |
| NT pro-BNP (pg/mL) | 609 (311, 1089) | 390 (213, 787) | **<0.001** |
| Sodium (mmol/L) | 139 (136, 141) | 139 (137, 141) | **<0.001** |
| Troponin T (ng/mL) | 0.03 (0.02, 0.09) | 0.03 (0.01, 0.03) | **<0.001** |
| **Vitals** |  |  |  |
| Body Mass Index (kg/m^2^) | 28.3 (23.4, 34.2) | 30.0 (25.1, 36.6) | **<0.001** |
| Weight (kg) |  |  |  |
| Weight Gain at Presentation | 0.0 (-4.1, 2.0) | 0.0 (-2.7, 2.2) | 0.134 |
| Weight Loss Over Hospitalization | 0.0 (-2.4, 2.7) | 0.0 (-0.6, 1.8) | **<0.001** |
| Weight Loss from Maximum | 2.5 (0.0, 6.8) | 1.6 (0.0, 4.8) | **<0.001** |
| Blood Pressure (mmHg) |  |  |  |
| Systolic BP- Admission | 131 (116, 149) | 135 (120, 152) | **0.030** |
| Systolic BP- Discharge | 123 (110, 141) | 129 (116, 143) | **<0.001** |
| Diastolic BP- Admission | 69 (60, 79) | 71 (62, 80) | **0.038** |
| Diastolic BP- Discharge | 66 (58, 75) | 70 (62, 78) | **<0.001** |
| Pulse Pressure- Admission | 62 (49, 77) | 63 (51, 77) | 0.175 |
| Pulse Pressure- Discharge | 57 (46, 70) | 59 (49, 70) | **0.029** |
| MAP- Admission (mmHg) | 92 (80, 102) | 92 (83, 103) | **0.033** |
| MAP- Discharge (mmHg) | 85 (76, 96) | 89 (82, 99) | **<0.001** |
| Heart Rate- Admission (mmHg) | 86 (73, 100) | 80 (70, 93) | **<0.001** |
| Heart Rate- Discharge (mmHg) | 80 (70, 92) | 75 (67, 83) | **<0.001** |
| **Medications** | | | |
| Aspirin | 304 (68.3) | 1049 (69.0) | 0.825 |
| ACE-I/ ARB/ ARNI | 223 (50.1) | 899 (59.1) | **0.001** |
| Aldosterone Antagonists | 24 (5.4) | 91 (6.0) | 0.723 |
| Beta Blocker | 366 (82.2) | 1150 (75.7) | **0.004** |
| Antiarrhythmics | 78 (17.5) | 165 (10.9) | **<0.001** |
| Anticoagulation | 116 (26.1) | 362 (23.8) | 0.362 |
| Ca-Channel Blocker (CCB), any | 156 (35.1) | 582 (38.3) | 0.237 |
| CCB, non-dihydropyridine | 113 (25.4) | 234 (15.4) | **<0.001** |
| Digoxin | 37 (8.3) | 50 (3.3) | **<0.001** |
| Diuretic- Metolazone | 46 (10.3) | 66 (4.3) | **<0.001** |
| Diuretic- Loop | 267 (60.0) | 759 (49.9) | **<0.001** |
| Diuretic- Thiazide | 63 (14.2) | 262 (17.2) | 0.143 |
| Pressor or Inotrope | 145 (32.6) | 284 (18.7) | **<0.001** |
| Nitrate | 60 (13.5) | 179 (11.8) | 0.375 |
| Hydralazine | 153 (34.4) | 483 (31.8) | 0.329 |
| Statin | 261 (58.7) | 924 (60.8) | 0.750 |
| Insulin | 238 (53.5) | 697 (45.9) | **0.005** |
| Metformin | 19 (4.3) | 125 (8.2) | **0.007** |
| Any Estrogen | 8 (1.8) | 26 (1.7) | 1.000 |
| NSAID | 51 (11.5) | 258 (17.0) | **0.006** |
| **Echocardiographic Findings** |  |  |  |
| Dilated LV | 21 (4.7) | 56 (3.7) | 0.395 |
| LV Diastolic Dysfunction | 346 (77.8) | 1084 (71.3) | **0.009** |
| LV Ejection Fraction | 59.8±5.7 | 59.9±5.4 | 0.562 |
| Dilated LA | 240 (53.9) | 735 (48.4) | **0.044** |
| Dilated RV | 85 (19.1) | 167 (11.0) | **<0.001** |
| RV Dysfunction | 58 (13.0) | 119 (7.8) | **0.001** |
| Pulmonary Hypertension | 191 (42.9) | 475 (31.2) | **<0.001** |
| Dilated Inferior Vena Cava | 48 (10.8) | 83 (5.5) | **<0.001** |
| Pericardial Effusion | 29 (6.5) | 72 (4.7) | 0.170 |
| MV E/A ratio | 1.1 (0.8, 1.5) | 1.0 (0.8, 1.4) | **<0.001** |
| *Indeterminate* | 95 (21.3) | 226 (14.9) |  |
| MV E/e' ratio | 14.5 (11.1, 19.8) | 13.8 (10.6, 18.6) | 0.127 |
| MV Peak E Wave, cm/s | 102.4 ± 34.2 | 96.4 ± 31.0 | **0.001** |
| MV Peak A Wave, cm/s | 88.4 ± 29.0 | 89.2 ± 29.0 | 0.613 |
| MV e' Velocity, cm/s | 7.1 ± 5.5 | 6.9 ± 3.4 | 0.495 |

Continuous variables are expressed as mean ± standard deviation. The categorical variables consisting of numerical values are expressed in terms of median (first quartile, third quartile). The remaining categorical variables are expressed in terms of n (%).

1. Comparisons between groups was performed using either a t-test or chi-square test. Variables with a p<0.10 (**bold**) were considered potentially significant and were selected further inclusion in machine-learning-based variable selection. [↑](#endnote-ref-1)
2. ICD-9/10 codes were used to identify noncompliance cases. [↑](#endnote-ref-2)
3. Acute HF on presentation is defined as either an ICD-9/10 code which denotes a secondary diagnosis of acute heart failure or an admission with a primary HF diagnosis or administration of intravenous diuretics during hospitalization with a secondary HF diagnostic codes of any acuity.

   ACE-I= angiotensin converting enzyme inhibitor; ARBs= angiotensin-receptor blockers; ARNI= angiotensin receptor-neprilysin inhibitor; BP= blood pressure; bpm= beats per minute; BUN= blood urea nitrogen; CABG= coronary artery bypass grafting; CCB= calcium channel blocker; COPD= chronic obstructive pulmonary disease; HF= heart failure; LV= left ventricular; MAP= mean arterial pressure; NSAID= non-steroidal anti-inflammatory drug; NT pro-BNP= N-terminal pro-brain natriuretic peptide; PCI= percutaneous coronary intervention; RV= right ventricle; TIA= transient ischemic attack. [↑](#endnote-ref-3)
