# Supplementary material for: Tailored risk assessment of 90‐day acute heart failure readmission or all‐cause death to heart failure with preserved versus reduced ejection fraction
Source: Clin Cardiol. 2022 Jan 25;45(4):370–8. doi: 10.1002/clc.23780 (PMC9019897; doi:10.1002/clc.23780)
Supplement: Supplementary file 3 — Supplementary information. [file CLC-45-370-s004.docx]

**Supplemental Table 3. Characteristics of HF with reduced EF based on 90-day acute heart failure readmission or all-cause death**

| **Characteristic** | **90-day HF event**  **(N=345)** | **No 90-day HF event**  **(N=879)** | **P-value^[[1]](#endnote-1)^** |
| --- | --- | --- | --- |
| **Demographic & Socioeconomic** | | | |
| Age (yr.) | 64.9 ± 15.1 | 68.4 ± 14.5 | **<0.001** |
| Male sex | 186 (53.9) | 527 (60.0) | **0.062** |
| Race |  |  | 0.950 |
| *Black* | 109 (31.6) | 270 (30.7) |  |
| *White* | 221 (64.1) | 569 (64.7) |  |
| *Other* | 15 (4.3) | 40 (4.6) |  |
| Married | 140 (40.6) | 372 (42.3) | 0.623 |
| Rural Resident | 297 (86.1) | 773 (87.9) | 0.433 |
| Insurance |  |  | **<0.001** |
| *Medicare* | 248 (71.9) | 517 (58.8) |  |
| *Medicaid* | 46 (13.3) | 105 (11.9) |  |
| *Private/Managed Care* | 8 (2.3) | 46 (5.2) |  |
| *Other* | 43 (12.5) | 211 (24.0) |  |
| **Outpatient Care** | | | |
| Electronic Health Portal Use | 61 (17.7) | 215 (24.5) | **0.013** |
| No. Cardiology Visits in 1-Year | 0 (0, 1) | 0 (0, 1) | **0.046** |
| No. No-Shows in 1-Year | 0 (0, 0) | 0 (0, 0) | **0.003** |
| **Social History** | | | |
| Tobacco Abuse/Smoking | 208 (60.3) | 512 (58.2) | 0.556 |
| Alcohol Dependence | 34 (9.9) | 94 (10.7) | 0.743 |
| Illicit Drug Use | 23 (6.7) | 63 (7.2) | 0.854 |
| Noncompliance^[[2]](#endnote-2)^ | 95 (27.5) | 247 (28.1) | 0.899 |
| **Medical History (Cardiovascular-related)** | | | |
| Hypertension | 317 (91.9) | 802 (91.2) | 0.804 |
| Dyslipidemia | 257 (74.5) | 621 (70.6) | 0.203 |
| Pulmonary Hypertension | 28 (8.1) | 38 (4.3) | **0.012** |
| Cardiomyopathy Diagnosis | 204 (59.1) | 556 (63.3) | 0.203 |
| Congestive Heart Failure | 204 (59.1) | 470 (53.5) | **0.084** |
| Coronary Artery Disease | 228 (66.1) | 567 (64.5) | 0.649 |
| Myocardial Infarction | 126 (36.5) | 308 (35.0) | 0.674 |
| CABG or PCI | 184 (53.3) | 430 (48.9) | 0.185 |
| Stroke or TIA | 184 (53.3) | 431 (49.0) | 0.197 |
| Pacemaker or Defibrillator | 44 (12.8) | 135 (15.4) | 0.284 |
| Valvular Heart Disease | 120 (34.8) | 250 (28.4) | **0.035** |
| Atrial Arrhythmia | 187 (54.2) | 420 (47.8) | **0.050** |
| Ventricular/Other Arrhythmia | 222 (64.3) | 476 (54.2) | **0.001** |
| Peripheral Arterial Disease | 102 (29.6) | 211 (24.0) | **0.053** |
| **Medical History (Other)** |  |  |  |
| Moderate/ Severe Renal Disease | 104 (30.1) | 241 (27.4) | 0.377 |
| Malignancy/ Cancer | 197 (57.1) | 486 (55.3) | 0.610 |
| Depression/ Psychiatric | 180 (52.2) | 436 (49.6) | 0.456 |
| . Cognitive Dysfunction | 50 (14.5) | 131 (14.9) | 0.926 |
| Diabetes | 175 (50.7) | 437 (49.7) | 0.799 |
| Endocrine- Thyroid Diseases | 101 (29.3) | 243 (27.6) | 0.617 |
| Hypogonadism | 19 (5.5) | 68 (7.7) | 0.214 |
| Venous Thromboembolism | 51 (14.8) | 93 (10.6) | **0.051** |
| Liver Disease | 108 (31.3) | 183 (20.8) | **<0.001** |
| Sleep Apnea | 103 (29.9) | 287 (32.7) | 0.381 |
| Lung Disease/ COPD | 205 (59.4) | 407 (46.3) | **<0.001** |
| Chronic Oxygen Use | 42 (12.2) | 67 (7.6) | **0.016** |
| Charlson Comorbidity Index | 3.5 ± 2.1 | 4.0 ± 2.1 | **<0.001** |
| **Hospitalization Characteristics** |  |  |  |
| Acute HF on Presentation^[[3]](#endnote-3)^ | 269 (78.0) | 519 (59.0) | **<0.001** |
| Length of Stay (days) | 6.9 ± 17.4 | 8.9 ± 8.9 | **0.009** |
| Observation Status | 336 (97.4) | 803 (91.4) | **<0.001** |
| Intensive Care Unit | 106 (30.7) | 201 (22.9) | **0.005** |
| Discharge Med Reconciliation | 36 (10.4) | 79 (9.0) | 0.502 |
| **Laboratory** |  |  |  |
| Albumin (g/dL) | 3.8 (3.3, 4.1) | 3.9 (3.6, 4.2) | **<0.001** |
| Bicarbonate (mmol/L**)** | 24.3 (21.6, 28.2) | 24.8 (22.0, 27.8) | **0.012** |
| BUN (mg/dL) | 24.2 (17.0, 37.2) | 18.8 (14.2, 26.7) | **<0.001** |
| Creatinine (mg/dL) | 1.2 (0.9, 1.6) | 1.0 (0.8, 1.4) | **<0.001** |
| Hemoglobin (g/dL) | 11.1 (9.3, 12.8) | 12.2 (10.5, 13.8) | **<0.001** |
| NT pro-BNP (pg/mL) | 570 (290, 899) | 542 (285, 1052) | **0.005** |
| Sodium (mmol/L) | 139 (136, 141) | 139 (137, 140) | **<0.001** |
| Troponin T (ng/mL) | 0.03 (0.02, 0.13) | 0.03 (0.02, 0.10) | **0.001** |
| **Vitals** |  |  |  |
| Body Mass Index (kg/m^2^) | 26.6 (22.7, 31.3) | 28.3 (24.2, 34.1) | <0.001 |
| Weight (kg) |  |  |  |
| Weight Gain at Presentation | 0.0 (-4.1, 0.7) | 0.0 (-2.9, 1.4) | **0.011** |
| Weight Loss Over Hospitalization | 0.0 (-1.6, 3.2) | 0.0 (-0.5, 3.0) | 0.701 |
| Weight Loss from Maximum | 2.9 (0.0, 6.7) | 2.2 (0.0, 5.4) | **0.096** |
| Blood Pressure (mmHg) |  |  |  |
| Systolic BP- Admission | 125 (110, 140) | 131 (117, 149) | **<0.001** |
| Systolic BP- Discharge | 116 (104, 132) | 123 (110, 137) | **<0.001** |
| Diastolic BP- Admission | 73 (64, 83) | 76 (67, 86) | **0.002** |
| Diastolic BP- Discharge | 68 (59, 76) | 71 (64, 79) | **<0.001** |
| Pulse Pressure- Admission | 51 (40, 65) | 54 (44, 66) | **0.004** |
| Pulse Pressure- Discharge | 48 (38, 60) | 50 (41, 62) | 0.391 |
| MAP- Admission (mmHg) | 90 (81, 101) | 95 (85, 106) | **<0.001** |
| MAP- Discharge (mmHg) | 84 (75, 94) | 89 (80, 98) | **<0.001** |
| Heart Rate- Admission (mmHg) | 92 (79, 104) | 87 (74, 100) | **0.007** |
| Heart Rate- Discharge (mmHg) | 81 (71, 92) | 76 (68, 85) | **<0.001** |
| **Medications** | | | |
| Aspirin | 272 (78.8) | 718 (81.7) | 0.290 |
| ACE-I/ ARB/ ARNI | 241 (69.9) | 696 (79.2) | **0.001** |
| Aldosterone Antagonists | 36 (10.4) | 85 (9.7) | 0.767 |
| Beta Blocker | 318 (92.2) | 831 (94.5) | 0.156 |
| Antiarrhythmics | 75 (21.7) | 126 (14.3) | **0.002** |
| Anticoagulation | 99 (28.7) | 239 (27.2) | 0.646 |
| Ca-Channel Blocker (CCB), any | 76 (22.0) | 204 (23.2) | 0.714 |
| CCB, non-dihydropyridine | 57 (16.5) | 149 (17.0) | 0.924 |
| Digoxin | 32 (9.3) | 49 (5.6) | **0.027** |
| Diuretic- Metolazone | 42 (12.2) | 37 (4.2) | **<0.001** |
| Diuretic- Loop | 216 (62.6) | 477 (54.3) | **0.010** |
| Diuretic- Thiazide | 35 (10.1) | 110 (12.5) | 0.291 |
| Pressor or Inotrope | 121 (35.1) | 158 (18.0) | **<0.001** |
| Nitrate | 52 (15.1) | 109 (12.4) | 0.250 |
| Hydralazine | 74 (21.4) | 214 (24.3) | 0.317 |
| Statin | 237 (68.7) | 615 (70.0) | 0.715 |
| Insulin | 188 (54.5) | 404 (46.0) | **0.009** |
| Metformin | 30 (8.7) | 70 (8.0) | 0.761 |
| Any Estrogen | 2 (0.6) | 13 (1.5) | 0.318 |
| NSAID | 53 (15.4) | 105 (11.9) | 0.131 |
| **Echocardiographic Findings** |  |  |  |
| Dilated LV | 92 (26.7) | 168 (19.1) | **0.005** |
| LV Diastolic Dysfunction | 331 (95.9) | 819 (93.2) | **0.090** |
| LV Ejection Fraction | 30.8 ± 9.8 | 27.4 ± 10.4 | **<0.001** |
| Dilated LA | 228 (66.1) | 497 (56.5) | **0.003** |
| Dilated RV | 93 (27.0) | 165 (18.8) | **0.002** |
| RV Dysfunction | 172 (49.9) | 310 (35.3) | **<0.001** |
| Pulmonary Hypertension | 141 (40.9) | 258 (29.4) | **<0.001** |
| Dilated Inferior Vena Cava | 51 (14.8) | 85 (9.7) | **0.014** |
| Pericardial Effusion | 18 (5.2) | 46 (5.2) | 1.000 |
| MV E/A ratio | 1.2 (0.9, 1.9) | 1.1 (0.8, 1.5) | **0.004** |
| *Indeterminate* | 35 (10.1) | 122 (13.9) |  |
| MV E/e' ratio | 16.9 (12.3, 24.3) | 14.8 (11.2, 20.9) | **0.002** |
| MV Peak E Wave, cm/s | 88.8 ± 29.4 | 95.8 ± 32.7 | **0.001** |
| MV Peak A Wave, cm/s | 77.7 ± 28.1 | 107.7 ± 578.0 | 0.336 |
| MV e' Velocity, cm/s | 5.9 ± 2.3 | 5.7 ± 3.6 | 0.231 |

Continuous variables are expressed as mean ± standard deviation. The categorical variables consisting of numerical values are expressed in terms of median (first quartile, third quartile). The remaining categorical variables are expressed in terms of n (%).

1. Comparisons between groups was performed using either a t-test or chi-square test. Variables with a p<0.10 (**bold**) were considered potentially significant and were selected further inclusion in machine-learning-based variable selection. [↑](#endnote-ref-1)
2. ICD-9/10 codes were used to identify noncompliance cases. [↑](#endnote-ref-2)
3. Acute HF on presentation is defined as either an ICD-9/10 code which denotes a secondary diagnosis of acute heart failure or an admission with a primary HF diagnosis or administration of intravenous diuretics during hospitalization with a secondary HF diagnostic codes of any acuity.

   ACE-I= angiotensin converting enzyme inhibitor; ARBs= angiotensin-receptor blockers; ARNI= angiotensin receptor-neprilysin inhibitor; BP= blood pressure; bpm= beats per minute; BUN= blood urea nitrogen; CABG= coronary artery bypass grafting; CCB= calcium channel blocker; COPD= chronic obstructive pulmonary disease; HF= heart failure; LV= left ventricular; MAP= mean arterial pressure; NSAID= non-steroidal anti-inflammatory drug; NT pro-BNP= N-terminal pro-brain natriuretic peptide; PCI= percutaneous coronary intervention; RV= right ventricle; TIA= transient ischemic attack. [↑](#endnote-ref-3)
