# Supplementary material for: Tailored risk assessment of 90‐day acute heart failure readmission or all‐cause death to heart failure with preserved versus reduced ejection fraction
Source: Clin Cardiol. 2022 Jan 25;45(4):370–8. doi: 10.1002/clc.23780 (PMC9019897; doi:10.1002/clc.23780)
Supplement: Supplementary file 4 — Supplementary information. [file CLC-45-370-s003.docx]

**Supplemental Table 4 AUCs from sensitivity analysis with different imputed values.**

| **Dataset** | **HFpEF**  **(Average; 95% CI)** | **HFrEF**  **(Average; 95% CI)** | **Entire cohort**  **(Average; 95% CI)** |
| --- | --- | --- | --- |
| Dataset 1 | 0.772 (0.768, 0.776) | 0.753 (0.748, 0.759) | 0.758 (0.755, 0.761) |
| Dataset 2 | 0.769 (0.765, 0.773) | 0.751 (0.745, 0.758) | 0.759 (0.755, 0.762) |
| Dataset 3 | 0.770 (0.766, 0.775) | 0.749 (0.743, 0.755) | 0.762 (0.759, 0.765) |
| Dataset 4 | 0.770 (0.766, 0.775) | 0.749 (0.743, 0.756) | 0.758 (0.754, 0.761) |
| Dataset 5 | 0.770 (0.766, 0.774) | 0.753 (0.747, 0.759) | 0.759 (0.756, 0.762) |

All the modeling procedures including variable selection and logistic regression were conducted with five different imputed values (denoted by dataset number).
